# Emerging Targets for Developing T Cell-Mediated Vaccines for Human Immunodeficiency Virus (HIV)-1

**DOI:** 10.3389/fmicb.2017.02091

**Published:** 2017-10-25

**Authors:** Danushka K. Wijesundara, Charani Ranasinghe, Branka Grubor-Bauk, Eric J. Gowans

**Affiliations:** ^1^Virology Laboratory, Basil Hetzel Institute for Translational Medicine, Discipline of Surgery, University of Adelaide, Adelaide, SA, Australia; ^2^Molecular Mucosal Vaccine Immunology Group, The John Curtin School of Medical Research, The Australian National University, Canberra, ACT, Australia

**Keywords:** human immunodeficiency virus, HIV-1, HIV vaccine, mucosal immunity, T cell, tissue resident memory, CD8^+^ T cell, vagina

## Abstract

Human immunodeficiency virus (HIV)-1 has infected >75 million individuals globally, and, according to the UN, is responsible for ~2.1 million new infections and 1.1 million deaths each year. Currently, there are ~37 million individuals with HIV infection and the epidemic has already resulted in 35 million deaths. Despite the advances of anti-retroviral therapy (ART), a cost-effective vaccine remains the best long-term solution to end the HIV-1 epidemic especially given that the vast majority of infected individuals live in poor socio-economic regions of the world such as Sub-Saharan Africa which limits their accessibility to ART. The modest efficacy of the RV144 Thai trial provides hope that a vaccine for HIV-1 is possible, but as markers for sterilizing immunity are unknown, the design of an effective vaccine is empirical, although broadly cross-reactive neutralizing antibodies (bNAb) that can neutralize various quasispecies of HIV-1 are considered crucial. Since HIV-1 transmission often occurs at the genito-rectal mucosa and is cell-associated, there is a need to develop vaccines that can elicit CD8^+^ T cell immunity with the capacity to kill virus infected cells at the genito-rectal mucosa and the gut. Here we discuss the recent progress made in developing T cell-mediated vaccines for HIV-1 and emphasize the need to elicit mucosal tissue-resident memory CD8^+^ T (CD8^+^ Trm) cells. CD8^+^ Trm cells will likely form a robust front-line defense against HIV-1 and eliminate transmitter/founder virus-infected cells which are responsible for propagating HIV-1 infections following transmission in vast majority of cases.

## Introduction

Since the discovery of human immunodeficiency virus (HIV)-1 as the causative agent of the acquired immunodeficiency syndrome (AIDS), significant progress has been made to thwart the worldwide spread of the virus despite many setbacks. The “Berlin Patient” highlighted that a cure for HIV/AIDS is possible through bone marrow transplantation (Hütter et al., 2009). However, the excessive cost of this procedure and the lack of compatible donors severely limit the application of this cure strategy. The use of anti-retroviral therapy (ART) remains the most feasible medical intervention to suppress the virus in patients. Furthermore, early treatment of infected individuals with ART can prevent HIV-1 transmission to uninfected individuals (Cohen et al., 2011) while the daily use of anti-retroviral drugs Truvada (i.e., for pre-exposure prophylaxis) by individuals can successfully prevent acquisition of HIV-1 (Molina et al., 2015). Unfortunately, it is estimated that more than half of the HIV-infected individuals are undiagnosed and 3/5 people infected with this virus do not have access to ART. This hinders the ability of ART to control the epidemic and given that ART does not eliminate latent reservoirs of HIV-1, strict and life-long adherence of ART is required for the therapy to be effective in patients.

A vaccine is essential to complement the efforts of ART to bring an end to HIV/AIDS especially in HIV endemic regions (e.g., Sub-Saharan Africa) manifested by the inability of patients to access ART adequately due to their poor socio-economic status. The main limitations to the development of an effective HIV-1 vaccine include the ability of the virus to mutate rapidly to facilitate immune escape and its ability to infect, deplete and establish viral reservoirs in CD4^+^ T cells. Despite these limitations, vaccines that elicit (i) broadly neutralizing antibodies (bNAb), (ii) non-neutralizing antibodies that direct virus-infected cells for destruction, or (iii) CD8^+^ T cell immunity have emerged as attractive targets for the future of HIV-1 vaccine development. In this brief review, we will discuss the avenues and challenges for developing an effective T cell-mediated vaccine for HIV-1 with a particular focus on CD8^+^ T cell immunity.

## Why do we need a vaccine which will elicit CD8^+^ T cell immunity?

There are several reasons which support the development of a vaccine to elicit CD8^+^ T cell immunity against HIV-1.

1. Elite controllers of HIV-1 and simian immunodeficiency virus (SIV) provide valuable clues for vaccine development because these individuals are able to suppress the virus to undetectable levels for prolonged periods in the absence of ART. Although enhanced activity of anti-viral CD8^+^ T cells is not always predictive of elite control of infection (Blankson, 2010), numerous studies in humans and macaques suggest that elite control strongly correlates with- or is dependent on- anti-viral CD8^+^ T cell responses that target conserved regions of SIV and HIV-1 proteins (Pontesilli et al., 1998; Saez-Cirion et al., 2007; Wijesundara et al., 2011; Chowdhury et al., 2015).
2. Anti-viral CD8^+^ T cells can directly kill virus-infected cells and can establish residency at sites where HIV-1 will likely be encountered during transmission (see below). Given that most HIV-1 infections are initiated by a single variant (i.e., transmitted/founder virus) (Zhu et al., 1993; Keele et al., 2008), the presence of HIV-specific CD8^+^ T cells at transmission sites will likely be crucial for reducing the viral set point and thereby reducing the reservoir size.
3. Passive transfer studies and vector-based immunoprophylaxis studies suggest that bNAb will be very effective in protection against HIV-1 (Balazs et al., 2011; Scheid et al., 2016; Caskey et al., 2017). Non-neutralizing antibodies and bNAb with the capacity to direct killing of cell-associated forms of HIV-1 have also been identified (Haynes et al., 2012; Malbec et al., 2013; Bruel et al., 2016). Some recent progress has also been made to develop a vaccine which will elicit bNAb (Pauthner et al., 2017). However, a significant conceptual breakthrough is still needed to develop a vaccination strategy to elicit high titres of potent bNAb and non-neutralizing antibodies especially at HIV-1 transmission sites. Furthermore, to be effective in preventing cell-to-cell transmission by eliminating cell-associated forms of the virus at transmission sites, antibody-based vaccine strategies will probably require sufficient numbers of innate effector cells (e.g., natural killer cells) to be present at transmission sites prior to a transmission event.
4. Therapeutic vaccines against HIV-1 and SIV have failed to demonstrate efficacy. However, a recent study demonstrated that a vaccination regimen which elicited broad CD8^+^ T cell immunity to SIV Gag, Pol, and envelope (Env) proteins protected SIV-infected macaques undergoing treatment interruption (Borducchi et al., 2016). Following on from this landmark study, a “kick and kill” approach (Shan et al., 2012) was exploited to increase the therapeutic efficacy of a recombinant modified vaccinia virus Ankara (MVA) vaccine in the ongoing BCN02-Romi (NCT02616874) proof of concept study. The findings presented by Beatriz Mothe at the 2017 Conference on Retroviruses and Opportunistic Infections in Seattle suggest that CD8^+^ T cell responses targeting conserved regions of HIV-1 were involved in protection.

## The importance of mucosal immunity for HIV-1 vaccine efficacy

HIV-1 is first encountered at the mucosa (genito-rectal tract), and the gastro-intestinal tract is the major site of HIV replication and CD4^+^ T cell depletion (Veazey and Lackner, 1998). It is well-established that a vaccine delivered mucosally can induce long-lasting immune responses at these mucosal sites, as systemic vaccination rarely induces optimal sustained mucosal immunity (Belyakov and Ahlers, 2009; Fouda et al., 2013; Sun et al., 2013). Mucosa-associated lymphoid tissues are linked to the “common mucosal immune system,” whereby antigenic stimulation at one site results in both local and distant mucosal immunity. According to the site of mucosal vaccination (i.e., oral, nasal, rectal, or vaginal), the immunity generated at different sites can be vastly different, mainly due to the unique antigen presentation mechanisms (which is dependent on the type of antigen presenting cells present at different antigen-sampling sites) and the homing markers they acquire at the local mucosa (Trivedi and Ranasinghe, 2015; Trivedi et al., 2015). For example, a vaccine delivered intranasally will induce mucosal immunity at the lung, genito-rectal tract and also the gastro-intestinal tract, whereas rectal delivery will only induce immunity in the rectum and the some instances in the intestine (Ranasinghe et al., 2011; De Rose et al., 2014; Tomusange et al., 2016a). However, different adjuvants and cytokines have the capacity to promote T cell migration to the mucosa by modulating the initial antigen presenting cell environment at the vaccination site (Xi et al., 2012; Trivedi et al., 2014). This may explain how some viral vectors (i.e., pox viral vector-based vaccines) delivered systemically can induce mucosal immunity (Stevceva et al., 2002; Marlin et al., 2017).

Despite that HIV-1 is transmitted mainly through mucosal surfaces, no mucosal vaccine has yet entered efficacy trials in humans, although a few phase I trials have been conducted (Leroux-Roels et al., 2013; Nyombayire et al., 2017). Studies in non-human primate models have demonstrated that mucosal CD8^+^ T cell responses can better control a mucosal viral challenge while systemic CD8^+^ T cell immunity is not fully effective against mucosal infection (Kent et al., 2005; Belyakov et al., 2006; Ferre et al., 2009). Several studies in animal models from our laboratories suggest that mucosal vaccination strategies can enhance the quality, viz. avidity and polyfunctionality, of HIV-specific CD8^+^ T cells and protection against surrogate HIV-1 challenge models (Ranasinghe et al., 2007, 2013, 2014). High quality CD8^+^ T cells can recognize and respond to low densities of peptide-major histocompatibility class I complexes, thereby eliminating infected cells at very early stages of infection (Alexander-Miller et al., 1996; Wijesundara and Ranasinghe, 2012). Several studies in humans suggest that high quality CD8^+^ T cell responses correlate with elite control of HIV-1 infection (Betts et al., 2006; Almeida et al., 2007; Berger et al., 2011). Consequently mucosal vaccination strategies are desirable to achieve protective outcomes against HIV-1 infections especially given that mucosal immunisations can elicit high quality mucosal CD8^+^ T cell responses. It is also likely that a mucosal vaccination regimen could be exploited to elicit tissue resident memory CD8^+^ T cells in the genito-rectal mucosa, which will likely form a front-line defense against HIV-1 as discussed below.

## Implications for eliciting tissue resident memory CD8^+^ T cells

The optimum protective outcomes following vaccination are achieved if immunity is established at sites where pathogens are initially encountered or migrate to in order to establish persistence or cause disease. Several recent discoveries in mice suggest that the presence of CD8^+^ tissue resident memory T (Trm) cells significantly enhance or are obligatory for protection against infections resulting from influenza virus, herpes simplex virus (HSV), vaccinia virus (VV), and *Plasmodium berghei* exposure (Fernandez-Ruiz et al., 2016; Mueller and Mackay, 2016). CD8^+^ Trm cells reside in tissues for the lifespan of the individual and the expression of CD69, CD103 (Mueller and Mackay, 2016), and/or high expression of CD11a (McNamara et al., 2017) are unique biomarkers that can be used to identify these cells in tissues of mice.

**Figure 1:**
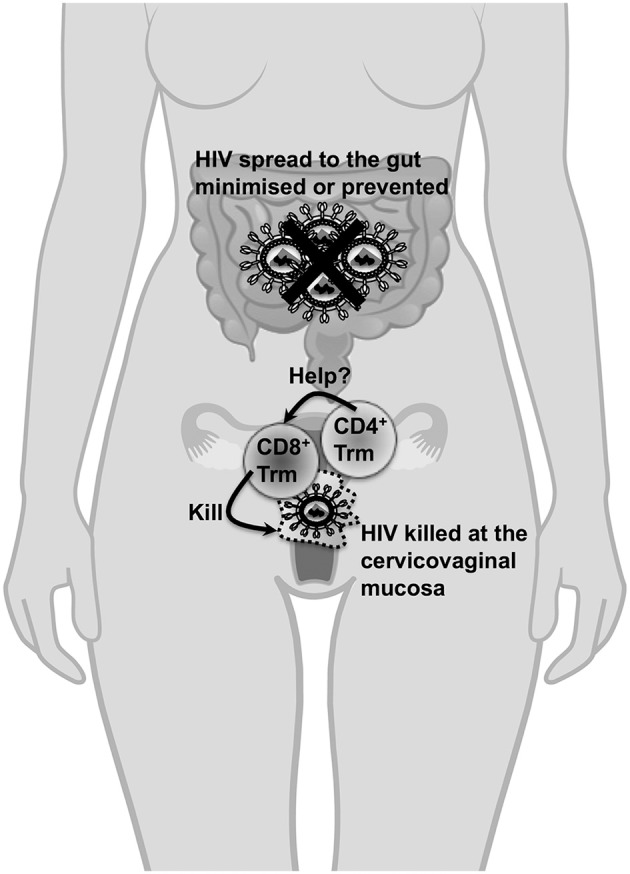
The importance of cervicovaginal Trm cells in protection against HIV-1. Cervicovaginal Trm cells will migrate through the cervicovaginal tissues for the lifespan of an individual and do not need to be recruited to the cervix or the vagina unlike other memory T cell subsets (which could take days to be recruited to these sites following primary exposure) during HIV-1 exposure. Thus, the presence of CD8^+^ Trm cells in cervicovaginal tissues is crucial to kill transmitted cell-associated forms of HIV and nascent infected cells resulting from cell-free virus during transmission. Most importantly, the transmitter/founder virus which initiates the HIV infection will likely be most vulnerable for destruction when CD8^+^ Trm cells are present at the cervicovaginal mucosa during primary exposure. Consequently, the likelihood of the virus spreading systemically, especially to the gut where a vast majority of CD4^+^ T cells are depleted during acute infection, is minimized or prevented. Furthermore, the presence of these Trm cells in this instance is also expected to significantly reduce the viral set point to an extent that it is unlikely for the infected individual to transmit the virus. It is unclear whether CD4^+^ Trm cells form in the cervicovaginal mucosa, whether they can act as targets for HIV infection or whether they will provide essential help to CD8^+^ Trm cells. The protective role of CD8^+^ Trm cells will likely apply to scenarios where HIV-1 is exposed in the rectum although there are no studies to demonstrate how these cells can be elicited in this site.

The vast majority of HIV-1 transmissions occur through sexual intercourse and heterosexual intercourse accounts for an estimated 70–80% of global HIV-1 transmissions. Thus, the appearance of HIV-specific CD8^+^ Trm cells in the rectal or cervicovaginal mucosa will likely contribute to protection during initial exposure to HIV-1 resulting from sexual transmission (Figure 1). The first study to describe the use of a vaccination regimen to elicit vaginal CD8^+^ Trm cells used a “prime and pull” strategy against HSV-2 (Shin and Iwasaki, 2012). The strategy involved priming the CD8^+^ T cells with a replication defective HSV-2 subcutaneously and then “pulling” the primed cells into the vaginal mucosa following intravaginal (i.vag) topical application of the CXCL9 and CXCL10. A few studies since have used i.vag delivery of human papillomavirus pseudovirions (HPV PsV) to induce the formation of cervicovaginal CD8^+^ Trm cells which correlated with protection against intravaginal challenge with VV and HSV (Çuburu et al., 2012, 2015). The cervicovaginal CD8^+^ Trm cells that were established following i.vag immunisations with HPV PsV express CD44, CD69, CXCR3, CD49a (α1 integrin), and CD103 (Çuburu et al., 2015).

These studies provide promise that vaccines can be exploited to elicit CD8^+^ Trm cells in the genito-rectal mucosa for protection against HIV-1 but several challenges remain in order to achieve this goal. Ideally, an HIV-1 vaccine will not require i.vag delivery as studies in macaques have highlighted that it will be difficult for vaccine vectors to penetrate the epithelium barrier of the vagina (Xu et al., 2013). Even in mice, i.vag delivery of HPV PsV required the use of progestins (Depo-Provera) to reduce the thickness of the vaginal epithelium and ensure PsV infection of cervicovaginal keratinocytes. Although a few recent studies suggest that expression of CD69 and CD103 could be used to characterize CD8^+^ Trm cells in the vagina of humans (Moylan et al., 2016; Posavad et al., 2017), the markers that uniquely represent these cells in the genito-rectal mucosa in macaques and humans are poorly characterized or unclear.

CD4^+^ T cell help allows dendritic cells to effectively prime effector CD8^+^ T cell responses and facilitate entry of effector CD8^+^ T cells into the female reproductive tract and ensure long-term survival of memory CD8^+^ T cells (Laidlaw et al., 2016). It is likely that a vaccine will need to elicit CD4^+^ T cell helper responses for efficient induction and survival of CD8^+^ Trm cells in cervicovaginal tissues. However, CD4^+^ T cells are major targets of HIV-1 infection and provision of cytokines such as interleukin-15 and type I interferons can be used to overcome the requirement of CD4^+^ T cell help for priming and survival of CD8^+^ T cells (Laidlaw et al., 2016). The presence/contribution of vaginal CD4^+^ Trm cells in protection against- or pathogenesis of- HIV infection is not well-understood (Figure 1). A better understanding of the requirement of CD4^+^ T helper cell responses for eliciting long-term CD8^+^ Trm cell responses in the reproductive tract and the rectum is necessary for rational HIV-1 vaccine design.

## Vectors and immunogens: lessons from humans and macaques

The failures of the only phase IIb/phase III trials (STEP, HVTN 503, and HVTN 505) to test the efficacy of a vaccine designed to elicit CD8^+^ T cells (Buchbinder et al., 2008; Hammer et al., 2013) have highlighted that the choice of vectors and immunogens are important determinants of vaccine efficacy in humans. In summary, the outcomes of these trials suggest that irrespective of the circumcision status of vaccine recipients and the presence of pre-existing immunity to Adenovirus serotype 5 (Ad5), the Ad5 vector used for vaccination is ineffective in humans. The exact reasons for the lack of efficacy in these trials is not known. However, eminent researchers have predicted that this could be due to the presence of Ad5-specific CD4^+^ T cells at mucosal sites (Barouch, 2010) and immunodominant CD8^+^ T cell responses directed to the highly variable Env protein (McMichael and Koff, 2014).

After the failures of the Ad5 vaccine, cytomegalovirus (CMV), Adenovirus serotype 26 (Ad26), Adenovirus serotype 35 (Ad35), and MVA vectors have emerged as extremely promising candidates for eliciting protective T cell responses against HIV-1. Vaccination of macaques with rhesus CMV encoding SIV Gag, Pol, Env, Rev, Nef, and Tat proteins provided superior and long-term control of SIV_mac251_ infection in 5/12 intra-rectally challenged animals (Hansen et al., 2011). Protection in this study was associated with robust effector memory CD8^+^ T cell responses and CD8^+^ T cell immunity targeting a broad-range of non-classical Major Histocompatibility Class-II restricted Gag epitopes (Hansen et al., 2011, 2013). Thus, although CMV may not represent an ideal vector with respect to safety of use, the strategy suggests that protection against persistent infection is possible, provided the CD8^+^ T cell responses are sufficiently robust and broad.

An alternative strategy that has gained considerable momentum since its development is the use of 2-valent mosaic antigens for vaccination (Barouch et al., 2010). The 2-valent mosaic antigens encompass native HIV Gag, Pol, and Env sequences that are most conserved amongst the isolates of clades B and C, and include as many CD4^+^ and CD8^+^ T cell epitopes as possible. Vaccines encoding mosaic antigens are capable of increasing the breadth of T cell responses to HIV Gag from clades A, B, and C whilst not compromising humoral immunity to Env from these clades (Barouch et al., 2010). Furthermore, vaccination of uninfected macaques with Ad26, Ad35, and MVA mosaic vaccines can elicit broad T cell responses and protective polyfunctional antibody responses especially when a liposome-based Env protein boost was used prior to SIV challenge (Barouch et al., 2010, 2013, 2015). Recently, a landmark therapeutic vaccination study exploited the Ad26 and MVA mosaic vaccines in a regimen involving a toll like receptor 7 agonist to increase the breadth of T cell responses to SIV Gag, Pol, and Env (Borducchi et al., 2016). In this study, the increased breadth of SIV-specific T cell immunity rather than humoral immunity correlated with partial protection and the regimen also allowed 3/9 vaccinated macaques to completely control SIV_mac251_ infection (Borducchi et al., 2016).

There may well also be a place for DNA vaccines especially given their recent success in the clinic (Trimble et al., 2015). We have recently shown that a novel cytolytic DNA vaccine which encodes a mutant form of perforin in addition to the immunogen is more immunogenic than a canonical DNA vaccine (Gargett et al., 2014). We have also shown that a DNA vaccine encoding Tat generated anti-Tat neutralizing antibodies (NAb) that inhibited the function of Tat in an *in vitro* transactivation assay (Tomusange et al., 2016b). As recent clinical trials reported that a clade B Tat vaccine induced cross-clade anti-Tat NAb in patients treated with ART, increased CD4^+^ T cell counts and reduced the viral load (Ensoli et al., 2016; Loret et al., 2016), some consideration should be given to combining a Gag/Pol/Env T cell vaccine with a vaccine designed to elicit anti-Tat NAb. Overall, the studies discussed above suggest that a vaccine vector designed to elicit protective T cell immunity against HIV-1 will need to encode as many conserved T cell immunogens of the virus as possible.

## Future directions

Although there are no correlates of protection from HIV-1, the induction of bNAb is considered to be the most desirable outcome for a HIV-1 vaccine, but a vaccine able to elicit such antibodies has not yet been developed for humans. However, many successful live attenuated virus vaccines (e.g., measles and yellow fever virus) that elicit protective NAb also elicit T cell immunity (Querec et al., 2006). HIV-1 employs multiple strategies to evade NAb (Schiffner et al., 2013) and cell to cell transmission is effective (Smith and Derdeyn, 2017). This suggests that an effective HIV-1 NAb vaccine regimen should also elicit CD8^+^ killer T cells, most likely against a broad range of highly conserved viral antigens as discussed previously. Thus, it is expected that a vaccine which will elicit heterotypic immunity, viz. bNAb and CD8^+^ T cell immunity, targeting conserved viral epitopes will be highly protective, but there are significant challenges to achieve this goal (Table 1). Perhaps the most urgent need with respect to a T cell-mediated vaccine is to develop a vaccine with the ability to not only induce systemic immune responses but also induce killer CD8^+^ T cell immunity at genito-rectal mucosa and the gut. In this regard, a vaccine with the capacity to elicit CD8^+^ Trm cells in the genito-rectal mucosa and the gut represents a plausible and a rational path for HIV-1 vaccine design in the future.

**Table 1:** Future challenges for the development of a T cell-mediated vaccine.

**Factor**	**Importance and caveats**
hCoice of immunogens	➢ It is important to encode as many immunogens as possible to elicit T cell immunity as broadly as possible to highly conserved regions of HIV-1 such as Gag and Pol. ➢ The caveat is that encoding variable regions of HIV-1 in a vaccine could drive immunodominant T cell responses to variable regions of the virus. Expansion of CD4^+^ T cell responses that target variable regions of HIV-1 could also contribute to diminishing the efficacy of a vaccine as they will be targets for infection.
Choice of vectors	➢ Replication-competent vectors that can induce cross-presentation in antigen presenting cells or replication incompetent (defective) vectors that can efficiently transduce antigen presenting cells are highly immunogenic. The chosen vector or a regimen involving several vectors should also be able to elicit long-lasting mucosal T cell immunity or Trm cells in the gut and the genito-rectal mucosa. ➢ The caveat is that replication competent vectors can have cytopathic effects deemed unsafe for use in humans and the insert capacity of a vector will also limit its ability to encode a broad range of immunogens. Vectors that can elicit Trm immunity in the vagina and the gut against SIV or HIV need to be developed and evaluated for protective efficacy.
Heterotypic immunity	➢ It is clear that a highly protective vaccination regimen will by necessity elicit heterotypic immunity (i.e., both T cell immunity and antibody responses especially bNAb with potent neutralization capacity). ➢ A vaccination strategy that elicits high titres of potent bNAb continues to represent a significant challenge and it is not clear how to develop a strategy that will elicit protective heterotypic immunity.
Translation	➢ Repetitive low-dose SIV challenge of vaccinated macaques is accepted as the best method to evaluate efficacy of a potential HIV-1 vaccine to be trialed in humans. ➢ Thus far the most robust T cell vaccine tested in macaques elicited control of SIV infection in 50% of the vaccinated macaques (Hansen et al., 2011). It is not clear whether this level of efficacy in macaques will translate to >50% efficacy in humans exposed to HIV-1 as this is a requirement to license a vaccine for use in humans.

## Author contributions

DW conceived the initial draft of the manuscript. CR, BG, and EG revised many parts of the manuscript, and contributed to finalize the manuscript.

### Conflict of interest statement

The authors declare that the research was conducted in the absence of any commercial or financial relationships that could be construed as a potential conflict of interest.
